# Surgical Excision of Heterotopic Ossification Leads to Re‐Emergence of Mesenchymal Stem Cell Populations Responsible for Recurrence

**DOI:** 10.5966/sctm.2015-0365

**Published:** 2016-10-05

**Authors:** Shailesh Agarwal, Shawn Loder, David Cholok, John Li, Chris Breuler, James Drake, Cameron Brownley, Joshua Peterson, Shuli Li, Benjamin Levi

**Affiliations:** ^1^ Burn/Wound and Regenerative Medicine Laboratory, Department of Surgery, University of Michigan, Ann Arbor, Michigan, USA

**Keywords:** Bone, Mesenchymal stem cell, Tissue regeneration, Chondrogenesis, Heterotopic ossification, Extremity trauma

## Abstract

Trauma‐induced heterotopic ossification (HO) occurs after severe musculoskeletal injuries and burns, and presents a significant barrier to patient rehabilitation. Interestingly, the incidence of HO significantly increases with repeated operations and after resection of previous HO. Treatment of established heterotopic ossification is challenging because surgical excision is often incomplete, with evidence of persistent heterotopic bone. As a result, patients may continue to report the signs or symptoms of HO, including chronic pain, nonhealing wounds, and joint restriction. In this study, we designed a model of recurrent HO that occurs after surgical excision of mature HO in a mouse model of hind‐limb Achilles’ tendon transection with dorsal burn injury. We first demonstrated that key signaling mediators of HO, including bone morphogenetic protein signaling, are diminished in mature bone. However, upon surgical excision, we have noted upregulation of downstream mediators of osteogenic differentiation, including pSMAD 1/5. Additionally, surgical excision resulted in re‐emergence of a mesenchymal cell population marked by expression of platelet‐derived growth factor receptor‐α (PDGFRα) and present in the initial developing HO lesion but absent in mature HO. In the recurrent lesion, these PDGFRα+ mesenchymal cells are also highly proliferative, similar to the initial developing HO lesion. These findings indicate that surgical excision of HO results in recurrence through similar mesenchymal cell populations and signaling mechanisms that are present in the initial developing HO lesion. These results are consistent with findings in patients that new foci of ectopic bone can develop in excision sites and are likely related to de novo formation rather than extension of unresected bone. Stem Cells Translational Medicine *2017;6:799–806*

**Significance Statement**

Secondary operations in common clinical scenarios such as joint replacements, traumatic wounds, and burn injuries are often complicated by a more robust inflammatory response, fibrosis, and complications such as heterotopic ossification than are primary operations. Heterotopic ossification (HO) presents a substantial barrier to rehabilitation of patients who have incurred severe trauma. However, surgical excision of HO is fraught with complications, including the development of recurrent ectopic bone. In this study, a model of recurrent HO similar to that seen in secondary procedures was validated, demonstrating that similar signaling mechanisms and mesenchymal cell populations responsible for initial HO are present within the recurrent HO lesion. These findings indicate that recurrent HO is not simply an extension of unresected bone but rather represents new heterotopic bone lesions for which prophylactic measures are likely required.

## Introduction

Trauma‐induced heterotopic ossification (HO) is the pathologic formation of extraskeletal bone that occurs after severe musculoskeletal injuries or orthopedic operations such as total hip arthroplasty (THA) [1, 2, 3, 4]. Interestingly, the incidence of HO in secondary THAs is nearly four times that of primary operations. HO insidiously develops in patients months after the inciting traumatic event. Patients may complain of chronic pain, the sensation of a bony prominence, loss of muscle or joint excursion, and even open wounds. When patients have these signs or symptoms, plain‐film radiographs or computed tomography (CT) imaging can elucidate the underlying cause.

**Figure 1:**
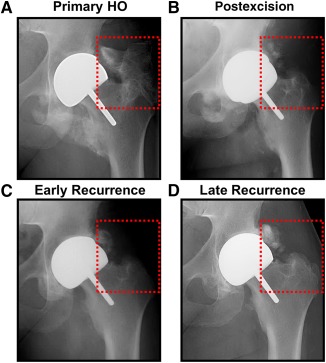
Clinical scenario of recurrent HO in a patient with total hip arthroplasty. **(A):** Primary HO in total hip arthroplasty patient (red square). **(B):** Postexcision radiograph showing substantial reduction in HO with some residual lesions. **(C):** Early evidence of recurrence after excision of HO. **(D):** Recurrent HO lesion 6 months after surgery at excision site. Abbreviation: HO, heterotopic ossification.

Currently, therapeutics targeted at HO are being investigated for their ability to prevent or mitigate the key signaling processes involved in the development of cartilage or subsequent ossification of this ectopic cartilage [5, 6, 7, 8]. However, patients who present with the signs or symptoms of HO typically already have ossified lesions with radiographic opacity (Fig. 1A). As a result, therapeutics targeting the step‐wise development of HO are ineffective, leaving surgical excision as the only viable option.

Surgical excision of HO is a challenging undertaking for several important reasons. First, it is unclear that excision of the offending bone will restore joint function or range of motion, reduce chronic pain, or even improve wound healing. Second, HO may develop near critical structures, including nerves, which precludes aggressive resection. As a result, HO resection may be incomplete. Finally, patients who undergo surgical excision have been noted to develop recurrences in the site of HO [9, 10, 11] (Fig. 1B–1D).

Recurrent HO is particularly difficult to manage because surgical excision is nearly impossible in a wound bed heavily scarred from the inciting trauma and subsequent initial HO excision. Additionally, patients who have undergone joint replacement surgery require aggressive rehabilitation to return to function, which is further impeded by the recurrent HO. Critical structures, including nerves or vessels, are difficult to visualize within the scarred tissue and may be destroyed during excision of recurrent HO, presenting limb‐ and even life‐threatening risk to the patient. However, it has been unclear whether HO recurrence is due to de novo ectopic cartilage forming secondarily to the repeated insult of surgery.

We previously developed a model of primary trauma‐induced HO, combining the systemic inflammatory insult of a dorsal skin burn with severe local connective tissue trauma in the form of an Achilles tenotomy. Although tenotomy in a mouse model has the capacity for limited HO formation, the addition of burn trauma generates robust and reliable formation of a primary HO lesion [2, 3]. In this study, we present our experience with a model of HO recurrence using a model of musculoskeletal trauma followed by excision and monitoring via microCT and histologic evaluation.

## Methods

### Mice

Animal procedures were carried out in accordance with published guidelines [12] approved by the Institutional Animal Care and University Committee on the Use and Care of Animals of the University of Michigan (UCUCA; approval no. PRO0005909). Wild‐type C57BL/6 male mice (age 8–10 weeks) were purchased from Charles River Laboratory (Wilmington, MA, http://www.criver.com) and used for all experiments. Mice were housed three per cage at 22°C ± 4°C (72°F ± 4°F) with 12 hours of light exposure daily (325 lux), and fed 5L0D chow (Land O’ Lakes, St. Louis, MO, http://www.labdiet.com).

### Burn Injury and Tenotomy Model for Initial HO Formation

All mice intended for in vivo development of heterotopic ossification underwent 30% total body surface area partial‐thickness burn injury to the dorsum under 3% inhaled isoflurane. A metal block was heated to 60°C in a water bath and placed over the shaved dorsum for 18 seconds. After burn injury, mice received a left hind‐limb Achilles tenotomy, using sterile surgical scissors, and the incision was closed with a single 5‐0 Vicryl suture. Animals were housed in UCUCA‐supervised facilities, three mice per cage, receiving 12 hours of light exposure daily, with no restrictions to diet. All mice were housed in the same facility.

### Model of Recurrent HO After Surgical Excision

The model of recurrent HO was performed by excision of HO that formed in the burn/tenotomy model. Nine weeks after burn/tenotomy injury, microCT imaging was performed to confirm presence of HO. The tenotomy site was re‐entered using a sterile scissors, and regions of ectopic bone disjoint from surrounding bony structures were excised under loupe magnification. Bone contiguous with the calcaneus was left undisturbed to avoid disruption of the normal bony cortex. The site was then closed with a single 5‐0 Vicryl suture.

### MicroCT Imaging and Analysis

In vivo development of HO was assessed with microCT scans 9 weeks after trauma, immediately before surgical excision (microCT using 80 kVp, 80 mA, and 1,100 millisecond exposure; GE Healthcare Biosciences, Pittsburgh, PA, http://www.gelifesciences.com). A subsequent microCT scan was performed within 3 days after excision to confirm the site of HO excision. A final microCT image was obtained 9 weeks after excision to re‐evaluate the site. Images were reconstructed and HO volume was computed using a calibrated imaging protocol [3]. The voxel size for all scans was 48.08 μm and the field of view was 38.47 cm × 38.47 cm. Scans were performed from the most distal aspect of the foot to the mid‐thigh. The region of interest evaluated for HO extended from the knee to the foot.

### Confirmation of Resection and De Novo HO Recurrence

Pre‐ and postsurgical microCT analysis of the excision site was performed along with a follow‐up microCT at 9 weeks postresection immediately before sacrificing the animal as described earlier in Methods. These serial CT images were used first to confirm the absence of residual bone at our resection site and second to guide further histologic analyses, as explained in detail later in this article.

We restricted our evaluation of recurrent HO to those de novo lesions occurring proximal to our resection site and that (a) did not have radiologic evidence of residual HO and (b) remained in discontinuity from any calcaneal remnant HO. We confirmed this first with CT imaging and then with follow‐up hematoxylin and eosin (H&E) staining of histologic sections to demonstrate the absence of osseous or cartilaginous tissue bridging any two distinct HO lesions.

### Histology

Animals were euthanized 3 weeks after the initial burn/tenotomy injury, 9 weeks after burn/tenotomy, or 9 weeks after surgical excision of HO. The injured hind limb was fixed in 10% formaldehyde at 4°C (39°F) for 24 hours, followed by decalcification in 19% EDTA. Specimens were then paraffin embedded and sliced 5‐μm thick. Slides were stained with H&E to obtain overall histologic architecture, aniline blue to assess mature osteoid, and safranin O to assess cartilage extracellular matrix. Immunostaining was performed using anti‐ bone morphogenetic protein‐2 (BMP2), anti‐pSMAD 1/5, anti‐collagen IIa (COLIIA), anti‐collagen X (COLX), anti‐Ki67, anti‐platelet‐derived growth factor receptor‐α (PDGFRα), and anti‐SOX9 (Santa Cruz Biotechnology, Santa Cruz, CA, https://www3.scbt.com).

## Results

### HO Recurred After Excision

Patients who undergo surgery for severe trauma injuries or joint replacement may undergo surgical excision to remove HO lesions. Mature, recurrent HO can be detected within the excisional wound bed with radiography (Fig. 1A–1D).

### Surgical Excision Resulted in HO Recurrence

**Figure 2:**
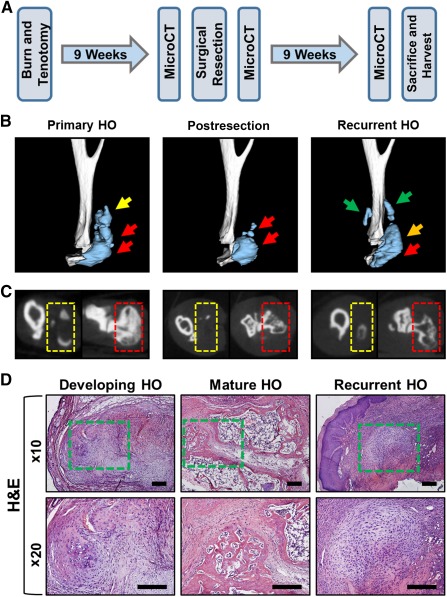
Recurrent HO following surgical excision. **(A):** Experimental design to develop recurrence. **(B):** Three‐dimensional microCT reconstructions showing images immediately prior to excision, immediately following excision, and recurrence 9 weeks after excision. Green arrows = site of de novo recurrent HO; orange arrow = site of HO developing from remnant HO; red arrows = site of original unresected HO; yellow arrow = region of resected HO. **(C):** MicroCT cross‐sections of HO lesions. Red box = site of unresected HO; yellow box = site of resected HO. **(D):** H&E staining showing architecture of early HO 3 weeks after injury, mature HO 9 weeks after injury, and recurrent HO 9 weeks after excision. Green box = site of HO. Abbreviations: H&E, hematoxylin and eosin; HO, heterotopic ossification; microCT, micro‐computed tomography.

Upon confirming that key mediators of initial HO development are absent after 9 weeks, we set out to create a model of HO recurrence. Therefore, 9 weeks after burn/tenotomy, mice underwent surgical excision of the identifiable HO lesions. MicroCT scans were performed immediately before and immediately after excision, followed by a subsequent microCT scan 9–12 weeks after excision (Fig. 2A). We found that the initial HO lesion could be excised surgically, albeit incompletely, based on pre‐ and immediate postexcision microCT imaging (Fig. 2B, 2C).

When microCT scans obtained immediately postexcision and at 9 weeks postexcision were compared, we noted interval increase in the size of the HO lesion, as well as the formation of distinct de novo osseous lesions occurring separately from the original site of HO formation (Fig. 2B, 2C). Based on these findings, we surmised that HO had recurred in and around the excision site. Histologic evaluation confirmed the presence of ectopic bone within the recurrent lesions after excision (Fig. 2D).

### Recurrent HO Lesions Showed Osteoid Characteristic of Early Initial HO Lesions

**Figure 3:**
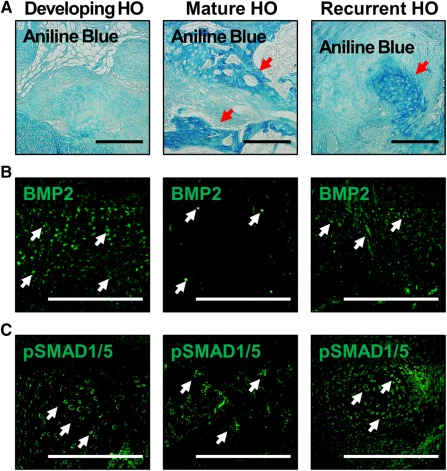
Osteoid and BMP signaling characteristics of recurrent HO. **(A):** Aniline blue staining shows presence of mature osteoid 9 weeks after HO, and emerging aniline blue staining in both early HO and developing recurrent HO lesions. Red arrows = sites of osteoid. **(B):** BMP2 expression is elevated in the developing HO lesion, reduced in mature osteoid 9 weeks after injury, and recurs in the recurrent HO lesion. **(C):** pSMAD 1/5 expression is elevated in the developing HO lesion, reduced in mature osteoid 9 weeks after injury, and recurs in the recurrent HO lesion. White arrows = representative sites of positive staining. Scale bar = 200 µm. Abbreviations: BMP2, bone morphogenetic protein‐2; HO, heterotopic ossification.

We set out, therefore, to confirm the presence de novo HO by using histologic analysis. To characterize the osteoid early after initial hind‐limb injury, tissue slices isolated from mice 3 weeks after injury were stained with aniline blue (Fig. 3A). These stains showed minimal evidence of mature osteoid. However, 9 weeks after burn/tenotomy, the entire heterotopic lesion consisted of mature osteoid with a developing marrow space. These findings established the character of osteoid within the initial HO lesion. Interestingly, when recurrent HO was analyzed 9 weeks after excision, the recurrent lesions showed regions with and without aniline blue stain (Fig. 3A). These findings suggest that recurrent HO exhibits characteristics of both mature and immature osteoid when analyzed 9 weeks postexcision.

### Recurrent HO Lesions Showed Evidence of Recurrent BMP Signaling

Bone morphogenetic protein (BMP) signaling has been implicated in the developing HO lesion, prompting our interest in whether this is apparent in the mature HO lesion 9 weeks after injury. Immunostaining for BMP2 and pSMAD 1/5, a downstream mediator of BMP signaling, was only minimally present in mature HO, in contrast with the developing lesion (Fig. 3B, 3C). These findings suggest that both BMP expression and downstream receptor signaling are substantially diminished in the mature HO lesion.

To demonstrate that recurrent HO lesions re‐express the key signaling mediators present in developing HO, we again performed immunostaining for BMP2 and pSMAD 1/5. Robust BMP2 and pSMAD 1/5 expression was observed in the recurrent HO lesion, consistent with the osteogenic tissue environment seen during early HO (Fig. 3B, 3C). Taken together, these findings confirm that recurrent HO lesions express signaling mediators present during the initial HO development but absent in mature HO.

### Recurrent HO Lesions Showed Evidence of New Cartilage

**Figure 4:**
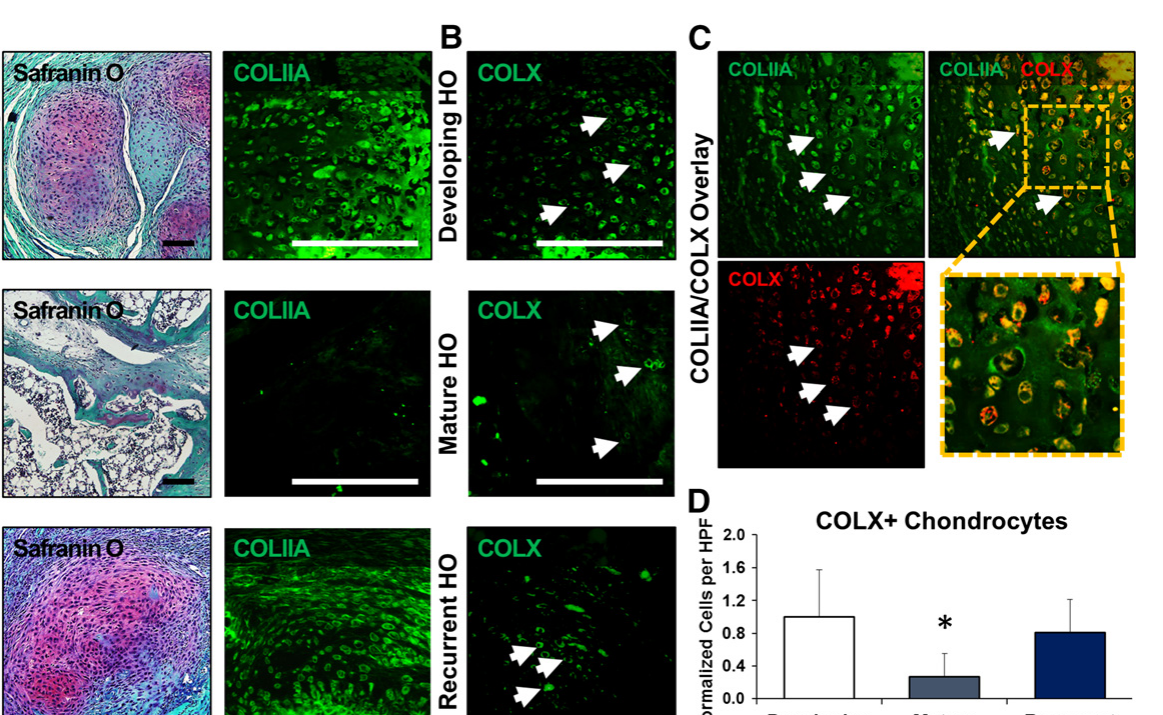
New cartilage in recurrent lesions undergoes de novo endochondral ossification. **(A):** Safranin O and COLIIA staining shows cartilaginous extracellular matrix in the developing and recurrent HO lesions, whereas mature HO shows marrow and osteoid with minimal cartilage presence. **(B):** Hypertrophic chondrocytes (white arrows) labeled by COLX immunostaining are present in developing HO and recurrent HO, but relatively diminished in mature HO. **(C):** Representative image demonstrating the overlap of COLIIA and COLX in hypertrophic chondrocytes (white arrows) present in areas of cartilage actively undergoing endochondral ossification. **(D):** Quantification of COLX+ hypertrophic chondrocytes in the cartilage anlagen of developing, mature, and recurrent HO. All values are normalized to developing HO. ∗, *p* > .05. Error bars represent standard deviation. Scale bars = 200 µm. Abbreviations: COLIIA, collagen II a; COLX, collagen X; HO, heterotopic ossification; HPF, high‐power field.

Because a substantial amount of the recurrent HO appeared immature, we next evaluated sections for cartilage using safranin O and COLIIA (Fig. 4A). Early after injury, characteristic safranin O and COLIIA staining was observed, which is indicative of cartilage presence. This staining pattern was no longer present 9 weeks after injury, consistent with the development of mature HO. However, 9 weeks after excision, new safranin O and COLIIA staining was observed within the re‐excision site. These findings are indicative of recurrent cartilage extracellular matrix formation following excision.

### Recurrent HO Lesions Underwent De Novo Endochondral Ossification

To confirm that cartilage present in recurrent lesions undergoes de novo endochondral ossification, we evaluated developing, mature, and recurrent HO using COLX. COLX expression in endochondral lesions is restricted to the hypertrophic chondrocytes that immediately proceed formation of ectopic bone. We found that COLX‐positive hypertrophic chondrocytes were present in both developing and recurrent HO (Fig. 4B). These cells were absent in the mature HO, consistent with the completion of endochondral ossification in those tissues (Fig. 4B). We further evaluated the overlap of COLIIA and COLX cells within developing mature HO and recurrent HO (Fig. 4C, 4D). Although we found no significant difference in the frequency of COLX‐positive chondrocytes (*p* = .45) between developing and recurrent lesions, mature lesions were significantly depleted of COLX‐positive cells (*p* = .009; Fig. 4D).

### Recurrent HO lesions Showed Evidence of Proliferative Mesenchymal Cells

**Figure 5:**
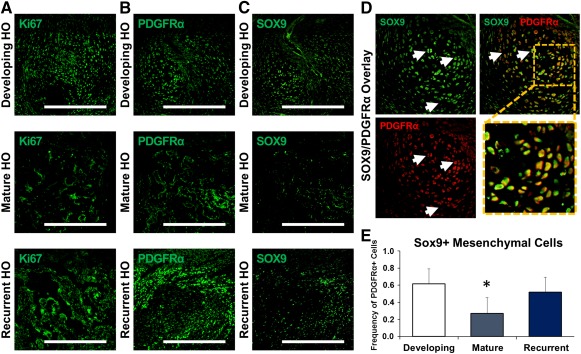
Contributing mesenchymal cell populations emerge in recurrent HO and undergo de novo chondrogenic differentiation. **(A):** Mesenchymal cell populations in the developing and recurrent HO are proliferative on the basis of Ki67 immunostaining. **(B):** Immunostaining for PDGFRα shows the presence of mesenchymal cells in developing HO and recurrent HO, with relative absence in mature HO. **(C):** Immunostaining for SOX9 shows presence of cells undergoing chondrogenic differentiation in developing HO and recurrent HO, with relative absence in mature HO. **(D):** Representative image demonstrating the overlap of SOX9 and PDGFRα (white arrows) in areas of mesenchymal cells actively undergoing chondrogenic differentiation. **(E):** Quantification of SOX9/PDGFRα costaining mesenchymal cells in developing, mature, and recurrent HO. ∗, *p* > .05. Error bars represent standard deviation. Scale bars = 200 µm. Abbreviations: HO, heterotopic ossification; PDGFRα, platelet‐derived growth factor receptor‐α.

To confirm a highly proliferative cellular population, immunostaining for Ki67 was performed (Fig. 5A). Ki67+ cells were absent in the mature osteoid but present in substantial quantities in both the developing HO lesion and the recurrent HO. To confirm that the new cartilage and bone being formed were not derived from the remaining unresected bone, immunostaining for mesenchymal cell populations was performed using PDGFRα (Fig. 5B). Immunostaining showed evidence of robust PDGFRα+ cell presence within the initial developing HO lesion. These cells were also positive for CD105 and CD90 (supplemental online Fig. 1). As expected, these undifferentiated mesenchymal cell populations were absent in the mature osteoid. However, within recurrent HO, PDGFRα+ re‐emerged similar to developing HO.

### Recurrent HO Lesions Showed Evidence of De Novo Chondrogenic Differentiation

To confirm that mesenchymal cells within the recurrent lesion were actually undergoing chondrogenic differentiation, immunostaining for the chondrogenic transcription factor SOX9 was performed (Fig. 5C). As expected, SOX9+ cells were present during the early phase of initial HO formation, as well as within recurrent lesions, which we had previously shown to be both safranin O and COLIIa positive (Fig. 4). There were few SOX9+ cells in the mature HO 9 weeks after injury (Fig. 5C). We then confirmed that these cells were indeed PDGFRα/SOX9 double positive via costaining (Fig. 5D, 5E). Notably, we found no significant difference in the extent of costaining between the developing primary and recurrent HO lesion (*p* = .29; Fig. 5E). Together, these findings indicate that recurrent HO forms through an endochondral intermediary and is not the result of direct extension from residual, unresected HO.

## Discussion

Trauma‐induced HO develops through an insidious process and typically presents with signs and symptoms including chronic pain and bony prominences, open wounds, and restricted range of motion and muscle excursion [1, 2, 3, 4]. When patients show radiographic evidence of HO, the only available recourse is surgical excision, which is unable to reverse the long‐term effects of ectopic bone formation, and which often results in recurrence. However, acute HO and recurrent HO are very different phenomena from the perspective of patient management. Whereas the initial HO excision may benefit from a relatively fresh wound bed with clear delineation of important structures (e.g., nerves or vessels), sites of recurrent HO are heavily scarred due to the initial trauma compounded by the subsequent excision. Additionally, recurrent HO can be psychologically difficult for patients who are already faced with intense rehabilitation regimens. Surgical re‐excision can risk loss of limb or life, or may again lead to recurrence or incomplete resection. Therefore, we must identify techniques that minimize the risk for recurrence in patients who have HO and desire surgical excision. This represents a highly targeted patient population, primed for postsurgical prophylactic intervention. In this study, we designed and validated a model of recurrent trauma‐induced HO that recapitulates critical signaling mediators involved in HO formation.

Initially, we found that although HO can recur as an extension of preexisting and remnant osseous tissue, de novo lesions form reliably both within the resection site and in adjacent tissues. These de novo lesions are radiographically and histologically distinct from areas of HO arising from preexisting bone and appear to undergo the same critical steps of HO formation that led to the primary lesion.

In particular, we found that during initial development, trauma‐induced HO sites showed high levels of both BMP2 and downstream BMP signaling mediators (e.g., pSMAD 1/5). BMP2 and pSMAD 1/5 staining is virtually absent when mature HO has formed, 9 weeks after the initial injury. However, surgical excision results in the redevelopment of these signaling factors with continued, long‐term expression. BMP signaling has previously been shown to be a mediator of heterotopic ossification, and is a known contributor to genetic forms of HO as well [5, 13, 14, 15]. These findings are consistent with the absence of cartilage on Safranin O staining when mature HO has formed, followed by renewed cartilage formation after excision. Heterotopic ossification is largely believed to occur through an endochondral process, and the identification of cartilage in recurrent lesions confirms that recurrent HO forms through a similar cartilage intermediary [16, 17, 18, 19]. Overall, these findings provide a mechanistic explanation for observed recurrence.

To exclude the possibility that the recurrence of HO was not simply interval growth of the previous HO, we performed immunostaining for cellular subtypes surrounding the recurrent ectopic bone, including chondrocytes (i.e., Sox9) and mesenchymal cells (i.e., PDGFRα). Both stains demonstrated evidence of cellular contributions to HO that extend beyond simple osteoid production, which would be implied by bone growth. These cell types are absent in mature HO before excision, suggesting that at least excision is leading to a resurgence of cell subtypes that eventually contribute to HO as well. Previous studies have indicated that PDGFRα+ mesenchymal cells are present in developing heterotopic ossification lesions [20, 21, 22, 23, 24]. These cells coexpress markers of chondrogenesis, including Sox9, and are believed to contribute to the growing ectopic cartilage lesion that eventually ossifies. In our model of recurrence, we identified these mesenchymal cells in the site of excision, which provides further evidence that our model is one of de novo ectopic bone rather than simple extension of unresected bone. Furthermore, these cells appear to be highly proliferative, consistent with their role in HO formation within the excision site.

This model of HO is easily reproducible and can be used by other researchers studying response to repeated trauma. First, the burn/tenotomy model was used to initiate HO formation at the site of tendon transection. Nine weeks later, the mature HO lesion was excised using a straight‐line vertical incision along the distal, posterior hind limb. The bone was visible under loupe magnification and easily removed from the surrounding soft‐tissue structures. Because the initial HO forms predictably in the burn/tenotomy model, surgical excision can be performed reliably. A critical aspect of this model is that microCT evaluation should be performed immediately prior to and following surgical excision to ensure that a portion or all of the bone was successfully excised. In our study, we found that a substantial amount of heterotopic bone could be excised but that some remnants of HO remained. However, the overwhelming presence of new PDGFRα+ cells and new cartilage formation based on safranin O and Sox9 staining confirmed that the new ectopic bone seen 9 weeks after excision was not simply an extension of unresected HO. Rather, it appears to be derived from mesenchymal cells that re‐emerge within the retraumatized excision site.

To our knowledge, this model appears to be the only one to demonstrate and validate recurrent heterotopic ossification. The clinical need for this model is clear based on radiographic evidence of recurrence, which occurs in 10%–15% of patients who undergo initial excision [25]. In a recent study of outcomes after surgical excision of HO, 6% of patients who underwent initial excision went on to require re‐excision surgery. In the face of a staggering number of patients with combat‐related injuries, joint replacement surgeries, or limb amputations who are at risk for HO, this represents a large absolute number of patients who may develop recurrence. Surprisingly, more than half of patients with radiographic evidence of recurrence were on a regimen of prophylactic measures consisting of nonsteroidal anti‐inflammatory drugs after excision [25]. These findings point to a need for improved prophylactic measures are based on an understanding of the mesenchymal cell populations and signaling processes involved in recurrence development.

Our findings are encouraging from a therapeutic perspective. They demonstrate similarities between recurrent HO and initial HO development. For patients who undergo surgical excision of HO, it is possible that therapeutics being evaluated for their ability to inhibit trauma‐induced HO initiation may also be used to prevent recurrence [6, 7, 26, 27, 28]. Prevention of recurrent HO presents a critical opportunity for physicians caring for patients postexcision. Although it can be difficult to determine whether a patient with traumatic injury may develop HO initially, it is easy to risk stratify patients who have already had HO and have undergone surgical excision. These patients are high risk, and treatment of all patients who have undergone surgical excision may represent an approach that can efficiently prevent HO.

The future value of this model lies not only with testing of therapeutics to demonstrate prevention of recurrence but also in studies to determine how new trauma can lead to bone growth. By identifying the cell subtypes responsible for new bone formation, we may be able to identify cells capable of healing normal bone defects.

## Author Contributions

S.A.: conception and design, collection and/or assembly of data, data analysis and interpretation, manuscript writing, final approval of manuscript; S. Loder: conception and design, collection and/or assembly of data, data analysis and interpretation, manuscript writing; D.C.: collection and/or assembly of data, data analysis and interpretation; J.L., C. Breuler, J.D., C. Brownley, J.P.: collection and/or assembly of data; S. Li: collection and/or assembly of data, data analysis and interpretation, final approval of manuscript; B.L.: conception and design, financial support, administrative support, provision of study material or patients, data analysis and interpretation, manuscript writing, final approval of manuscript.

## Disclosure of Potential Conflicts of Interest

The authors indicated no potential conflicts of interest.

## Supporting information

Supporting Information
