# Supplementary material for: Surgical Excision of Heterotopic Ossification Leads to Re‐Emergence of Mesenchymal Stem Cell Populations Responsible for Recurrence
Source: Stem Cells Transl Med. 2016 Oct 5;6(3):799–806. doi: 10.5966/sctm.2015-0365 (PMC5442786; doi:10.5966/sctm.2015-0365)
Supplement: Supplementary file 1 — Supporting Information [file SCT3-6-0799-s001.pdf]

Supplemental Figure – Levi et al.

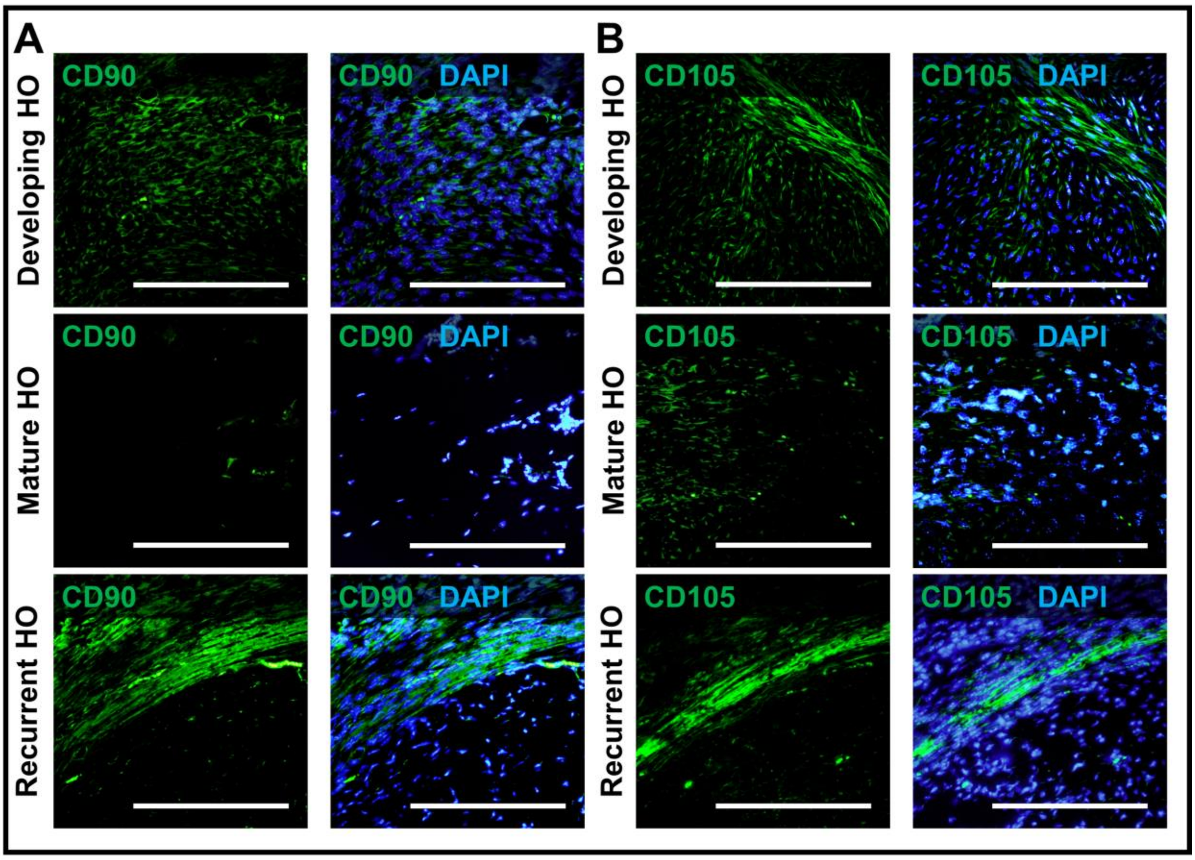

Fig S1. Characterization of mesenchymal cell populations demonstrates similarities between developing and recurrent HO and undergo *de novo* chondrogenic differentiation. (A) Immunostaining for CD90 shows presence of mesenchymal cells in developing HO and recurrent HO, with relative absence in mature HO; (B) Immunostaining for CD105 shows presence of mesenchymal cells in developing HO and recurrent HO, with relative absence in mature HO. Scale Bars represents 200 µm.
